# Structural brain abnormalities in 12 persons with aniridia

**DOI:** 10.12688/f1000research.11063.2

**Published:** 2017-09-01

**Authors:** Madison K. Grant, Anastasia M. Bobilev, Jordan E. Pierce, Jon DeWitte, James D. Lauderdale

**Affiliations:** 1 Department of Cellular Biology, University of Georgia, Athens, GA, 30602, USA; 2 Neuroscience Division of the Biomedical and Health Sciences Institute, University of Georgia, Athens, GA, 30602, USA; 3 Department of Psychology, University of Georgia, Athens, GA, 30602, USA; 4 Athens Radiology Associates, Athens, GA, 30604, USA

**Keywords:** MRI, PAX6, neuroanatomy

## Abstract

**Background:** Aniridia is a disorder predominately caused by heterozygous loss-of-function mutations of the *PAX6* gene, which is a transcriptional regulator necessary for normal eye and brain development.  The ocular abnormalities of aniridia have been well characterized, but mounting evidence has implicated brain-related phenotypes as a prominent feature of this disorder as well.  Investigations using neuroimaging in aniridia patients have shown reductions in discrete brain structures and changes in global grey and white matter.  However, limited sample sizes and substantive heterogeneity of structural phenotypes in the brain remain a challenge. **Methods:** Here, we examined brain structure in a new population sample in an effort to add to the collective understanding of anatomical abnormalities in aniridia.  The current study used 3T magnetic resonance imaging to acquire high-resolution structural data in 12 persons with aniridia and 12 healthy demographically matched comparison subjects. **Results:** We examined five major structures: the anterior commissure, the posterior commissure, the pineal gland, the corpus callosum, and the optic chiasm.  The most consistent reductions were found in the anterior commissure and the pineal gland; however, abnormalities in all of the other structures examined were present in at least one individual. **Conclusions:** Our results indicate that the anatomical abnormalities in aniridia are variable and largely individual-specific.  These findings suggest that future studies investigate this heterogeneity further, and that normal population variation should be considered when evaluating structural abnormalities.

## Introduction

Aniridia is panocular, congenital, and progressive disorder with an occurrence of approximately 1 in 83,000 live births ^1, 2^. Aniridia is best characterized by the lack of or hypoplasia of the iris (for which it is named), in addition to several other ocular abnormalities, which culminate in reduced visual acuity ^3^. Due to the progressive nature of the disease, individuals usually develop multiple ocular abnormalities, such as keratopathy, corneal vascularization and opacification, glaucoma, anterior chamber fibrosis, and cataracts ^4– 7^. Although aniridia is most well known for its ocular phenotypes, the condition has a number of other abnormalities, including sensory, neural, cognitive, and auditory processing abnormalities ^8, 9^.

The development of aniridia in humans is linked to heterozygous loss-of-function mutations to the *PAX6* gene, which encodes a highly conserved transcription factor critical for normal eye and neural development ^1^. The vast majority of aniridia cases (80%) are associated with mutations in *PAX6* ^2, 10^. Functional mutations in this gene can be either sporadic or familial, and causal mutations in aniridia encompass a large number of variants. The majority of these variants are nonsense mutations, which lead to a premature termination codon, and are found across the *PAX6* locus ^10, 11^. *PAX6* is expressed in the developing eye, brain, and spinal cord, and is required for aspects of anatomical and functional development of the central nervous system (CNS) and visual system ^12^. Within the CNS, *PAX6* is involved in patterning, regionalization, and the formation of neural circuits ^13– 16^. Previous studies of patients with aniridia using structural magnetic resonance imaging (MRI) have shown abnormalities in major fiber tracts and subcortical structures of the brain, including the anterior commissure ^9, 17– 20^, posterior commissure ^18, 20^, corpus callosum ^9, 19, 20^, pineal gland ^18, 20^, optic chiasm ^20^, and olfactory bulb ^17, 18^. The most consistently reported abnormalities are found in the anterior commissure, pineal gland, and optic chiasm; abnormalities in the posterior commissure, corpus callosum, and olfactory bulb are found in fewer than 35% of patients examined. Additionally, studies have shown conflicting results regarding grey matter volume differences in aniridia, with reports of both increases and decreases in whole brain grey matter ^21, 22^. Most recently, it has been shown that there is an accelerated age-related increase in cortical thinning of the inferior parietal lobe and prefrontal/premotor areas in both brain hemispheres in aniridia compared to healthy patients ^23^.

While several previous studies have investigated structural brain abnormalities in patients with aniridia, the variance in brain structures affected and extent of anatomical abnormalities is high. The variance observed in the published literature may be interpreted as a result of genetic differences in patient samples, either directly related to disease-causing mutations or modifier effects caused by genomic differences across subjects. Most of the previous studies examining structural changes in the brain of aniridia patients have focused on a subset of the structures we examined, but only one other study has looked at all five together ^20^. Additionally, we used a 3T magnet instead of a 1.5T, which allows for higher resolution structural images of small structures such as interhemispheric commissures, allowing us to more reliably identify subtle difference. The current study sought to investigate gross anatomical correlates of aniridia in a new population sample with varied *PAX6* mutations. Results from this study will serve as a comparison for previous studies, as well as contribute to what is known about the distribution of neuroanatomical phenotypes in the aniridia population as a whole. Overall, this will serve to clarify the extent of abnormalities in five brain structures in persons with aniridia with varied mutations to the *PAX6* gene and contribute to our global understanding of the neuroanatomical characteristics of the disorder.

## Methods

### Subjects

A total of 14 individuals with aniridia and 15 healthy comparison individuals participated in the current study. Data from two participants with aniridia were excluded from analyses (due to a significant artifact and missing data). One healthy subject was excluded due to an anatomical abnormality, and two others were excluded because they did not match the demographic profile of an individual in the aniridia group included in the analysis. The remaining 12 individuals with aniridia (7 females; 3 left handed; mean age=36 years, SD=15) and 12 age- and gender-matched comparisons (7 females; 4 left handed; mean age=35 years, SD=14) were included in the analyses ( Table 1). Healthy comparison subjects were recruited through flyers posted in the community. Participants with aniridia were recruited through the Aniridia Foundation International Conference held in 2011 in Athens, Georgia and had been clinically diagnosed with aniridia. Exonic sequencing of the *PAX6* gene (11p13) (OMIM: 607108) was conducted at the University of Georgia, as previously described ^10, 24^. All mutations, which can be found in Table 1, have been submitted to the Human *PAX6* Allelic Variant Database ( http://lsdb.hgu.mrc.ac.uk/home.php?select_db=PAX6), as part of a previous genotype identification study ^10^. Three of the participants with aniridia belonged to the same family, and all other participants included in the analyses were unrelated. After written informed consent was obtained and MRI safety screening was conducted, all participants completed an MRI session in which a high-resolution structural scan was obtained. The Institutional Review Board of the University of Georgia approved all activities prior to subject recruitment, data collection, and data analysis (project number: 2011-10862-1; STUDY00003122).

**Table 1.** ANIRIDIA. Subject demographics and structural abnormalities as seen in structural magnetic resonance images. Gender, age, handedness, mutation, and structural abnormalities of both aniridia subjects and healthy comparisons. Subject ID: Numbers are matched subjects, A refers to aniridia subjects, C refers to healthy comparisons. ND, not determined.

ANIRIDIA										
Subject ID	Gender	Age	Handedness	Mutation	Predicted Mutation Effect	Anterior Commissure	Posterior Commissure	Pineal Gland	Corpus Callosum	Optic Chiasm
1A	Male	18	Right	c.949C>T	Nonsense	Reduced	Normal	Reduced	Normal	Reduced
2A	Female	19	Ambidextrous	c.771delG	Frameshift deletion	Reduced	Normal	Reduced	Slightly reduced	Reduced
3A ^*^	Male	20	Right	c.204delC	Frameshift deletion	Reduced	Normal	Reduced	Normal	Reduced
4A ^*^	Female	24	Left	c.204delC	Frameshift deletion	Reduced	Reduced	Reduced	Normal	Reduced
5A	Female	25	Left	ND	ND	Reduced	Reduced center	Highly reduced	Normal	Reduced
6A	Female	28	Right	c.28C>T	Nonsense	Reduced	Normal	Absent	Slightly reduced	Normal
7A	Male	39	Right	c.482delG	Frameshift deletion	Reduced	Normal	Reduced	Normal	Normal
8A	Male	47	Right	ND	ND	Reduced	Normal	Reduced	Normal	Normal
9A ^*^	Male	46	Left	c.204delC	Frameshift deletion	Reduced	Normal	Reduced	Normal	Reduced
10A	Female	51	Right	c.766-3C>G	Splice junction disruption	Reduced	Reduced	Absent	Normal	Normal
11A	Female	53	Right	ND	ND	Reduced	Reduced	Reduced	Normal	Normal
12A	Female	60	Ambidextrous	c.799A>T	Nonsense	Reduced	Reduced	Reduced	Slightly reduced	Reduced

* Indicates individuals from a single family

**Table:** 

PAX6 NORMAL										
Subject ID	Gender	Age	Handedness	Mutation	Predicted Mutation Effect	Anterior Commissure	Posterior Commissure	Pineal Gland	Corpus Callosum	Optic Chiasm
1C	Male	18	Right	*PAX6* Normal	N/A	Normal	Normal	Normal	Normal	Normal
2C	Female	20	Right	*PAX6* Normal	N/A	Normal	Normal	Normal	Normal	Normal
3C	Male	21	Right	*PAX6* Normal	N/A	Normal	Normal	Slightly Reduced	Normal	Normal
4C	Female	22	Left	*PAX6* Normal	N/A	Normal	Normal	Normal	Normal	Normal
5C	Female	23	Left	*PAX6* Normal	N/A	Normal	Normal	Normal	Normal	Normal
6C	Female	28	Left	*PAX6* Normal	N/A	Normal	Normal	Normal	Normal	Normal
7C	Male	37	Right	*PAX6* Normal	N/A	Normal	Normal	Normal	Normal	Normal
8C	Male	50	Right	*PAX6* Normal	N/A	Normal	Normal	Normal	Normal	Normal
9C	Male	50	Left	*PAX6* Normal	N/A	Reduced	Normal	Normal	Normal	Normal
10C	Female	48	Right	*PAX6* Normal	N/A	Reduced	Reduced	Absent	Normal	Normal
11C	Female	51	Right	*PAX6* Normal	N/A	Normal	Normal	Reduced	Normal	Normal
12C	Female	56	Right	*PAX6* Normal	N/A	Normal	Normal	Reduced	Normal	Normal

### MRI data acquisition

All data were collected on a 3T GE Signa MRI (General Electric, Milwaukee, WI, USA) at the University of Georgia’s Bio-Imaging Research Center. To obtain a high-resolution structural scan, images were acquired with a T1-weighted 3D FSPGR sequence [echo time (TE)=min full, flip angle=20°; field of view (FOV)=240 mm × 240 mm; matrix size=256 × 256, 150 axial slices; in-slice resolution=0.94 × 0.94 mm; slice thickness=1.2 mm].

### MRI structural analysis

MR images were transferred to a DICOM image format and analyzed using two software programs, SPM run on MATLAB and OsiriX. For SPM analysis, DICOM files were converted to nifti format and analyzed using Statistical Parametric Mapping Software (SPM8; Wellcome Trust Centre for Neuroimaging; http://www.fil.ion.ucl.ac.uk/spm/) run on a MATLAB software platform (MATLAB Release 2015b; The Mathworks, Inc., Natick, MA, USA). SPM software was used to compare aniridia subjects to their demographically matched comparison subjects. DICOM files were additionally analyzed using Osirix Lite DICOM viewer (OsiriX v5.6,;Pixmeo SARL, Bernex, Switzerland) and all images generated using this software. All 24 individual subjects’ data were visually inspected for gross anatomical abnormalities in two independent sessions by the first author (MG) and a radiologist (JDW). Regions of interest were determined by literature review to include the anterior commissure, posterior commissure, pineal gland, corpus callosum, and optic chiasm. The only structure that has been examined in the literature that we did not examine in our population was the olfactory bulb. This structure was excluded because both evaluators independently determined that the olfactory bulb could not be reliably assessed in the current data set. The radiologist was blinded to patient status and genotype during visual examination of scans. No new regions of interest were determined during both the first and second examination of the scans.

### Corpus callosum quantitative analysis

Following the approach of Free and colleagues (2003), the cross-sectional area of the corpus callosum was quantified at the mid-sagittal plane ^21^. In order to align all MR images to the mid-sagittal plane, individual T1 images were reconstructed in AFNI software ^25^ and aligned manually using the anterior commissure, posterior commissure, and interhemispheric landmarks. On the mid-sagittal image, the corpus callosum was manually traced and the number of voxels within this delineation was counted. Additionally, the structural volumes were automatically segmented with FreeSurfer software ( http://surfer.nmr.mgh.harvard.edu) to obtain estimates of total grey and white matter volume ^26, 27^. The ratio of corpus callosum area to total cerebral volume was calculated to control for differences in overall brain size (see Supplementary Figure 1 “Ratio”). Supplementary Figure 1 graphs were created using GraphPad Prism 7 software ( https://www.graphpad.com/).

## Results

**Figure 1.**
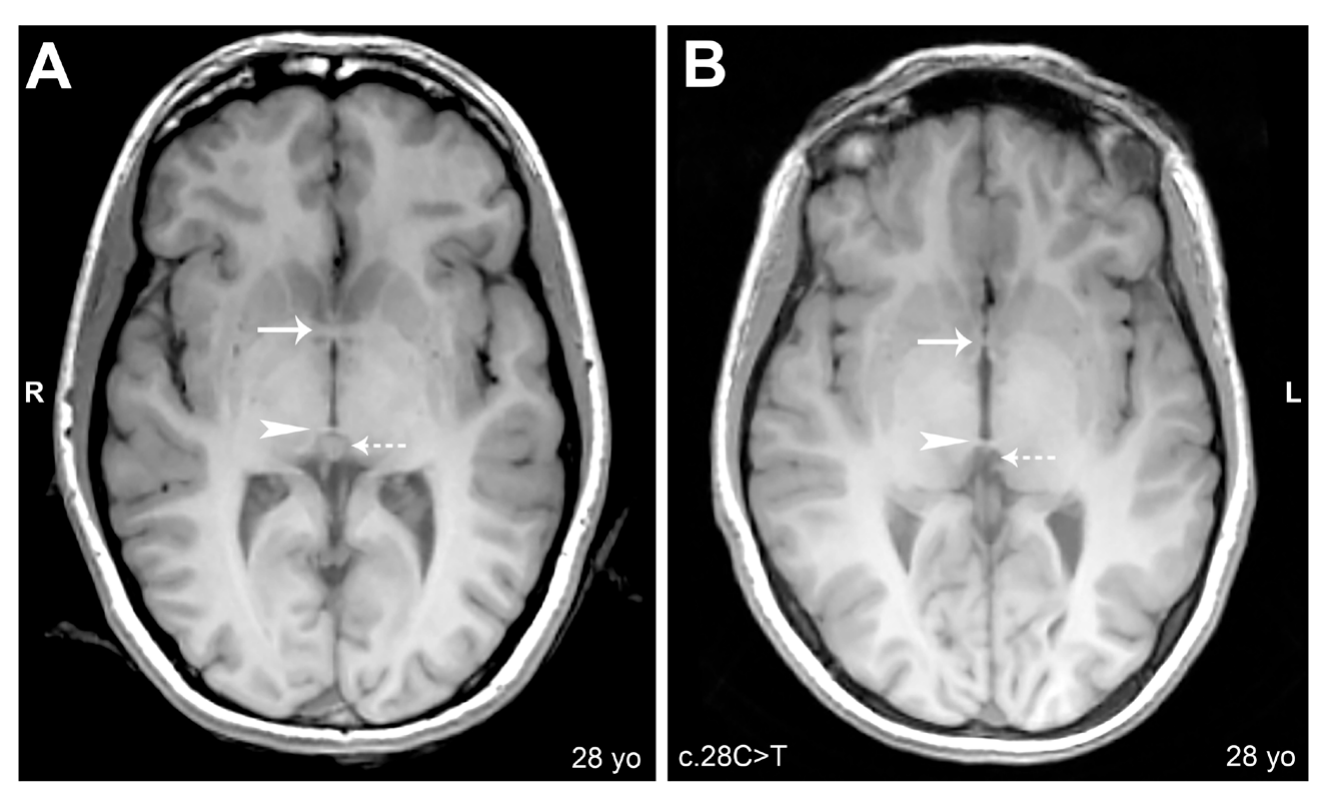
Anterior commissure, posterior commissure, and pineal gland: Axial cerebral T1-weighted magnetic resonance images, slice thickness 1.2mm. ( **A**) Subject 6C: Arrow shows normal anterior commissure; arrowhead shows normal posterior commissure; dashed arrow shows normal pineal gland. ( **B**) Subject 6A: Arrow shows reduced anterior commissure; arrowhead shows normal posterior commissure; dashed arrow shows absence of pineal gland.

**Figure 2.**
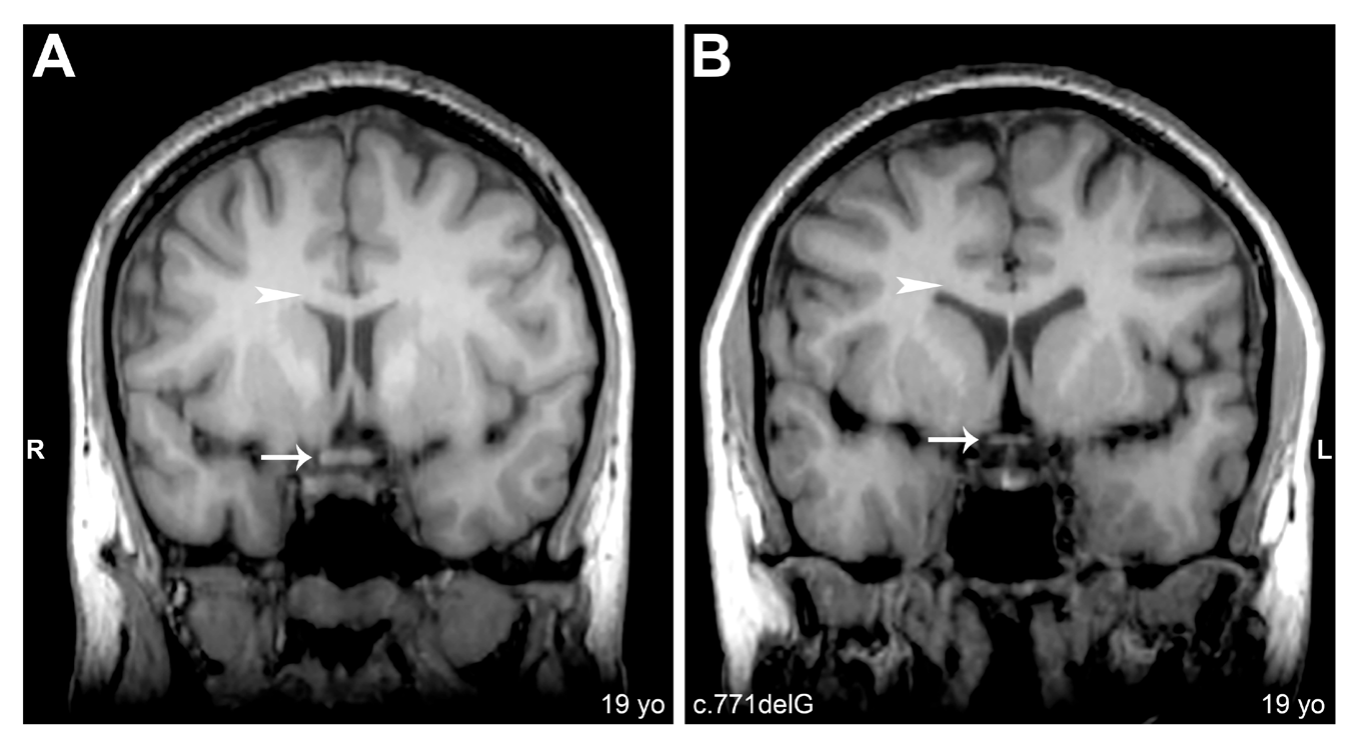
Optic chiasm and corpus callosum: Coronal cerebral T1-weighted magnetic resonance images, slice thickness 1.2mm. ( **A**) Subject 2C: Arrow shows normal optic chiasm. ( **B**) Subject 2A: Arrow shows reduced optic chiasm. Arrowheads in **A** and **B** denote normal corpus callosum.

Whole brain analysis was used to identify major structural abnormalities in aniridia patients. All neuroanatomical results are reported in Table 1, which includes subject demographics and mutations. We analyzed five major brain structures that demonstrate clear anatomical abnormalities in aniridia patients. Our study showed that all 12 aniridia patients had a reduced anterior commissure when compared to their demographically matched healthy comparisons, as shown in Figure 1. The posterior commissure was reduced in 5/12 aniridia patients and normal in 7/12 of aniridia patients ( Figure 1). Of the five aniridia patients with reduced posterior commissures, one had a reduced commissure at the midline. The pineal gland was affected in all 12 aniridia patients: absent in two ( Figure 1), highly reduced in one, and reduced in nine. The corpus callosum was slightly thinned in 3/12 aniridia patients and normal in 9/12 aniridia patients ( Figure 2). The optic chiasm was reduced in 7/12 of patients ( Figure 2) and normal in 5/12 patients.

In an effort to consider normal structural variation in the healthy population, we also evaluated healthy comparisons for structural brain abnormalities. We saw a reduced anterior commissure in two of the healthy comparisons and a reduced posterior commissure in one healthy comparison. The pineal gland showed the most variance within the healthy comparison group with one subject with no visible pineal gland, three healthy comparisons with slightly reduced to reduced pineal glands, and eight healthy comparisons with normal pineal glands. All healthy comparisons had normal corpus callosums and optic chiasms. A full description of which structures showed abnormalities in both the aniridia and healthy comparison groups are reported in Table 1. These findings provide context for asserting disease-related changes in the aniridia population, in the current study as well as others.

Additionally, we examined corpus callosum area at the mid-sagittal plane along with grey matter, white matter, and whole brain volume in our aniridia and healthy comparison groups. Grey matter volume and total brain volume averages are indistinguishable between aniridia and healthy comparison subjects. There is a slight reduction in the aniridia patients compared to healthy individuals in average corpus callosum area as well as white matter volume and ratio of corpus callosum area to whole brain volume. However, these are just trends and no measurement was statistically significant, which can also be explained by a high degree of variability within the aniridia population. Volumetric and area measurements along with graphical representations of the data can be seen in Supplementary Table 1 and Supplementary Figure 1.

Aniridia and healthy comparison MRI data Structural MRI DICOM files for 12 aniridia and 12 healthy comparison individuals. Files are in DICOM format and labeled according to subject I.D. found in Table 1. These files can be opened using SPM software run in Matlab or OsiriX DICOM viewer (see Methods section). Click here for additional data file. Copyright: © 2017 Grant MK et al. 2017 Data associated with the article are available under the terms of the Creative Commons Zero "No rights reserved" data waiver (CC0 1.0 Public domain dedication).

## Discussion

The most commonly reported neuroanatomical abnormality in MRI studies of aniridia patients is the anterior commissure. Previous studies have reported changes in the anterior commissure with some cases described as reductions and others reported as complete absence of the structure ^9, 17– 19^. Consistent with these reports, our study identified a reduction in the anterior commissure in all 12 aniridia patients, but none of the patients lacked the structure. The posterior commissure has also been evaluated in previous studies: one study reports that it is present in all subjects while the other study presented evidence that one patient had an absent structure while the others had normal posterior commissures ^18, 20^. We found no individuals lacking the posterior commissure, and fewer than half of our aniridia patients seemed to have an abnormal structure. Interestingly though, it seems as if one patient exhibited a reduction of the posterior commissure at the midline with thickened bundles adjacent to the midline suggesting that commissure formation was incomplete. PAX6 has a known role in formation of the posterior commissure in rodents, so it is likely that this phenotype in humans is a direct consequence of PAX6 deficiency ^15^. In agreement with previous findings, we also see abnormal or absent pineal glands in our entire patient population. This finding is consistent with sleep regulation deficits in persons with aniridia reported in other studies ^28^. The corpus callosum has also been a structure commonly evaluated in aniridia MRI studies, with many reporting reductions in corpus callosum thickness and severe agenesis ^9, 19, 20^. However, we found very few patients present with a reduced or abnormal corpus callosum, and propose that the slight reduction we see in three of our patients falls within normal population variation. Unlike most previous studies, we examined the optic chiasm, and found a reduction in the structure in more than half of our patients. This reduction could be a developmental consequence of the disorder or a progressive phenotype associated with reduced levels of PAX6. Anatomical abnormality findings seem to be highly dependent on population sample, and a larger collective sample in the literature will help us get closer to understanding common disease traits and variation. 

As described above, we observed a reduction in the anterior commissure in every patient we evaluated and a reduction of the posterior commissure in five patients, but no individuals completely lacked either structure. Our study utilized a high resolution 3T MRI for data acquisition, while most other studies used a 1.5T MRI. Signal to noise ratio from 3T MRIs are almost double that of 1.5T MRIs, which will lead to an improved image quality and resolution from the 3T magnet ^29^. We propose that the difference in observing a reduced versus absent anterior/posterior commissure may be due to a difference in scan resolution between images captured from a 3T versus 1.5T magnet. We see multiple patients in our group who have a severely reduced anterior commissure, and identifying this abnormality using a 1.5T magnet may be more difficult than when using a 3T. Additionally, the posterior commissure is smaller than the anterior commissure naturally, making it more difficult to distinguish between presence and absence in a scan. Lower scan resolution may not capture small structures such as these commissures, especially if they are reduced in size, leading to a false judgment of their absence. 

A recent study has found an age component to cortical thickness in aniridia patients when compared to healthy individuals. The study found that in patients with aniridia there is an accelerated reduction in cortical thickness of the inferior parietal lobe and prefrontal/premotor areas in both brain hemispheres ^23^. Adding to this age component seen in the Yogarajah (2016) study, there are also population differences in brain anatomy, even within healthy groups, between younger subjects and older subjects ^30^. Additionally, as we show in our study, there are anatomical abnormalities even within healthy, unaffected participants. This makes it vitally important for careful selection of comparison subjects, and presents a caveat for interpreting differences we see in this and other clinical populations. 

In addition to abnormal structural findings in patient populations with aniridia, multiple studies have assessed volumetric differences in grey and white matter in the brains of aniridia versus healthy comparisons. Similar to the findings in gross structural differences, much variation exists between reports. Some studies show an increase in grey matter volume in aniridia patients compared to healthy control groups, while others find both increases and decreases depending on brain region ^21, 22^. Changes in white matter findings follow the same suit with some reports of reductions in white matter and others finding both reductions and increases ^21, 22^. Even more interestingly, structures, such as the anterior commissure, posterior commissure, and pineal gland, show no deviation from healthy in these group-wise comparisons. This suggests that either the abnormalities seen in these structures are not as common among aniridia patients as previously thought, or that the characteristics of anatomical changes observed in aniridia patients have a high degree of variability within the population. Due to the consistency of abnormalities in structures like the anterior commissure in our study, as well as others, we predict the latter explains this discrepancy. This explanation is supported by the consistency of our results from volumetric and visual inspection of the corpus callosum in the current dataset. It is also possible that the differences observed across and within studies of these structures are a result of scan resolution and inconsistency of structure localization or plane of imaging to capture them given their small size and overall individual anatomical variation. 

## Conclusions

The current study investigated anatomical brain abnormalities correlated to aniridia in a new population sample in an effort to serve as a comparison to previous studies. Our aim was to contribute to what is known about the distribution of neuroanatomical phenotypes in the aniridia population as a whole. Although we found similar neuroanatomical abnormalities as previous studies, we find the severity is not as great as previously reported. The anterior commissure and pineal gland seem to be the structures most affected in the aniridia patients we examined, and we do see abnormalities in the posterior commissure, corpus callosum and the optic chiasm, albeit at lower frequency than previously reported. We believe the neuroanatomical abnormalities seen in aniridia populations have a high level of variability, and future studies should be aimed at collecting more patient MRI scans so that the breadth of abnormalities can be assessed. 

## Ethical approval and consent

MRI sessions were completed after written informed consent was obtained and MRI safety screening was completed. All activities were approved by the Institutional Review Board of the University of Georgia prior to subject recruitment and data collection. All individuals who participated in this study provided consent for their demographic, mutation information, and images to be published.

## Data availability

The data referenced by this article are under copyright with the following copyright statement: Copyright: © 2017 Grant MK et al.

Data associated with the article are available under the terms of the Creative Commons Zero "No rights reserved" data waiver (CC0 1.0 Public domain dedication). 

**Dataset 1: Aniridia and healthy comparison MRI data:** Structural MRI DICOM files for 12 aniridia and 12 healthy comparison individuals. Files are in DICOM format and labeled according to subject I.D. found in Table 1. These files can be opened using SPM software run in Matlab or OsiriX DICOM viewer (see Methods section). doi, 10.5256/f1000research.11063.d153799 ^31^

Mutation information that has been presented here is also available through *PAX6* Allelic Variant Database ( http://lsdb.hgu.mrc.ac.uk/home.php?select_db=PAX6).

## Figures and Tables

**Supplementary Table 1.** Subject corpus callosum area and brain volume measurements. Grey matter volume, white matter volume, total brain volume, and corpus callosum area of aniridia and matched subjects. Ratio refers to corpus callosum area/total brain volume. Subject ID: Numbers are matched subjects, A refers to aniridia subjects, C refers to healthy comparisons.

ANIRIDIA					
Subject ID	Grey Matter Volume (mm ^3^)	White Matter Volume (mm ^3^)	Total Volume (mm ^3^)	Corpus Callosum Area (mm2)	Ratio: CC area/total volume
1A	795957	654158	1450115	774	0.000534
2A	711626	604800	1316426	552	0.000419
3A ^*^	683393	585559	1268952	712	0.000561
4A ^*^	568045	505360	1073405	584	0.000544
5A	595209	573664	1168873	648	0.000554
6A	543481	496139	1039620	542	0.000521
7A	602438	545307	1147745	566	0.000493
8A	646003	616039	1262042	732	0.00058
9A ^*^	621706	607971	1229677	684	0.000556
10A	533370	495092	1028462	713	0.000693
11A	623640	592662	1216302	652	0.000536
12A	528675	489748	1018423	555	0.000545

* Indicates individuals from a single family

**Table:** 

PAX6 NORMAL					
Subject ID	Grey Matter Volume (mm ^3^)	White Matter Volume (mm ^3^)	Total Volume (mm ^3^)	Corpus Callosum Area (mm2)	Ratio: CC area/total volume
1C	653601	568784	1222385	685	0.00056
2C	600216	547621	1147837	653	0.000569
3C	710079	668109	1378188	657	0.000477
4C	611409	542922	1154331	643	0.000557
5C	605760	545683	1151443	883	0.000767
6C	626978	587857	1214835	963	0.000793
7C	728278	734113	1462391	667	0.000456
8C	632857	647710	1280567	721	0.000563
9C	595440	613750	1209190	477	0.000394
10C	523144	484070	1007214	618	0.000614
11C	667226	616716	1283942	795	0.000619
12C	525662	540868	1066530	856	0.000803

**Supplementary Table 2.** Subject ID. Subject ID used in this paper, MRI DICOM ID for DICOM file matching, and Person ID from *Bobilev et al. 2016* ^10^.

Subject ID	MRI DICOM ID	*Bobilev et al.* *2016* Person ID
1A	50009	1001A316/sUGA
2A	50005	124A141/s-124
3A	50022	147A061/f-149
4A	50013	147A064/f-148
5A	50014	N/A
6A	50036	157A121/s-157
7A	50003	114A159/f-173
8A	50010	N/A
9A	50012	147A017/f-147
10A	50015	173A291/s-220
11A	50011	N/A
12A	50016	191A297/s-222
1C	1002	N/A
2C	50031	N/A
3C	1003	N/A
4C	50028	N/A
5C	50044	N/A
6C	50037	N/A
7C	50003 Match	N/A
8C	50034	N/A
9C	1017	N/A
10C	1070	N/A
11C	50032	N/A
12C	50064	N/A

## Supplementary Material

**Supplementary Figure 1: Corpus Callosum Cross-sectional area and brain volume comparisons.** ( **A**) Representation of manually traced corpus callosum in a mid-sagittal slice. ( **B**) Corpus callosum area. ( **C**) Grey matter volume. ( **D**) White matter volume. ( **E**) Total brain volume. ( **F**) Ratio of corpus callosum area to total brain volume. Line in each graph represents average of group. No statistically significant differences were observed for any of the measurements depicted in this figure (p < 0.05).

## References

[1] Hanson IM Seawright A Hardman K: PAX6 mutations in aniridia. *Hum Mol Genet.* 1993;2(7):915–920. 10.1093/hmg/2.7.915 8364574

[2] Grønskov K Olsen JH Sand A: Population-based risk estimates of Wilms tumor in sporadic aniridia. A comprehensive mutation screening procedure of PAX6 identifies 80% of mutations in aniridia. *Hum Genet.* 2001;109(1):11–18. 10.1007/s004390100529 11479730

[3] Nelson LB Spaeth GL Nowinski TS: Aniridia. A review. *Surv Ophthalmol.* 1984;28(6):621–42. 10.1016/0039-6257(84)90184-X 6330922

[4] Grant WM Walton DS: Progressive changes in the angle in congenital aniridia, with development of glaucoma. *Am J Ophthalmol.* 1974;78(5):842–847. 10.1016/0002-9394(74)90308-0 4423758

[5] McCulley TJ Mayer K Dahr SS: Aniridia and optic nerve hypoplasia. *Eye (Lond).* 2005;19(7):762–764. 10.1038/sj.eye.6701642 15359227

[6] Nishida K Kinoshita S Ohashi Y: Ocular surface abnormalities in aniridia. *Am J Ophthalmol.* 1995;120(3):368–375. 10.1016/S0002-9394(14)72167-1 7661209

[7] Tsai JH Freeman JM Chan CC: A progressive anterior fibrosis syndrome in patients with postsurgical congenital aniridia. *Am J Ophthalmol.* 2005;140(6):1075–1079. 10.1016/j.ajo.2005.07.035 16376654

[8] Thompson PJ Mitchell TN Free SL: Cognitive functioning in humans with mutations of the *PAX6* gene. *Neurology.* 2004;62(7):1216–1218. 10.1212/01.WNL.0000118298.81140.62 15079031

[9] Bamiou DE Musiek FE Sisodiya SM: Deficient auditory interhemispheric transfer in patients with *PAX6* mutations. *Ann Neurol.* 2004;56(4):503–509. 10.1002/ana.20227 15389894

[10] Bobilev AM McDougal ME Taylor WL: Assessment of PAX6 alleles in 66 families with aniridia. *Clin Genet.* 2016;89(6):669–77. 10.1111/cge.12708 26661695 PMC4873406

[11] Hingorani M Williamson KA Moore AT: Detailed ophthalmologic evaluation of 43 individuals with *PAX6* mutations. *Invest Ophthalmol Vis Sci.* 2009;50(6):2581–90. 10.1167/iovs.08-2827 19218613

[12] Kim J Lauderdale JD: Analysis of Pax6 expression using a BAC transgene reveals the presence of a paired-less isoform of Pax6 in the eye and olfactory bulb. *Dev Biol.* 2006;292(2):486–505. 10.1016/j.ydbio.2005.12.041 16464444

[13] Manuel M Price DJ: Role of Pax6 in forebrain regionalization. *Brain Res Bull.* 2005;66(4–6):387–393. 10.1016/j.brainresbull.2005.02.006 16144620

[14] Simpson TI Price DJ: Pax6; a pleiotropic player in development. *Bioessays.* 2002;24(11):1041–1051. 10.1002/bies.10174 12386935

[15] Mastick GS Davis NM Andrew GL: Pax-6 functions in boundary formation and axon guidance in the embryonic mouse forebrain. *Development.* 1997;124(10):1985–1997. 9169845 10.1242/dev.124.10.1985

[16] Osumi N: The role of Pax6 in brain patterning. *Tohoku J Exp Med.* 2001;193(3):163–174. 10.1620/tjem.193.163 11315763

[17] Sisodiya SM Free SL Williamson KA: PAX6 haploinsufficiency causes cerebral malformation and olfactory dysfunction in humans. *Nat Genet.* 2001;28(3):214–216. 10.1038/90042 11431688

[18] Mitchell TN Free SL Williamson KA: Polymicrogyria and absence of pineal gland due to *PAX6* mutation. *Ann Neurol.* 2003;53(5):658–663. 10.1002/ana.10576 12731001

[19] Bamiou DE Free SL Sisodiya SM: Auditory interhemispheric transfer deficits, hearing difficulties, and brain magnetic resonance imaging abnormalities in children with congenital aniridia due to PAX6 mutations. *Arch Pediatr Adolesc Med.* 2007;161(5):463–469. 10.1001/archpedi.161.5.463 17485622

[20] Abouzeid H Youssef MA ElShakankiri N: *PAX6* aniridia and interhemispheric brain anomalies. *Mol Vis.* 2009;15:2074–83. 19862335 PMC2765237

[21] Free SL Mitchell TN Williamson KA: Quantitative MR image analysis in subjects with defects in the *PAX6* gene. *Neuroimage.* 2003;20(4):2281–2290. 10.1016/j.neuroimage.2003.07.001 14683729

[22] Ellison-Wright Z Heyman I Frampton I: Heterozygous PAX6 mutation, adult brain structure and fronto-striato-thalamic function in a human family. *Eur J Neurosci.* 2004;19(6):1505–12. 10.1111/j.1460-9568.2004.03236.x 15066147

[23] Yogarajah M Matarin M Vollmar C: *PAX6*, brain structure and function in human adults: advanced MRI in aniridia. *Ann Clin Transl Neurol.* 2016;3(5):314–30. 10.1002/acn3.297 27231702 PMC4863745

[24] Pierce JE Krafft CE Rodrigue AL: Increased functional connectivity in intrinsic neural networks in individuals with aniridia. *Front Hum Neurosci.* 2014;8:1013. 10.3389/fnhum.2014.01013 25566032 PMC4271605

[25] Cox RW: AFNI: software for analysis and visualization of functional magnetic resonance neuroimages. *Comput Biomed Res.* 1996;29(3):162–73. 10.1006/cbmr.1996.0014 8812068

[26] Dale AM Fischl B Sereno MI: Cortical surface-based analysis. I. Segmentation and surface reconstruction. *Neuroimage.* 1999;9(2):179–194. 10.1006/nimg.1998.0395 9931268

[27] Fischl B Salat DH Busa E: Whole brain segmentation: automated labeling of neuroanatomical structures in the human brain. *Neuron.* 2002;33(3):341–55. 10.1016/S0896-6273(02)00569-X 11832223

[28] Hanish AE Butman JA Thomas F: Pineal hypoplasia, reduced melatonin and sleep disturbance in patients with *PAX6* haploinsufficiency. *J Sleep Res.* 2016;25(1):16–22. 10.1111/jsr.12345 26439359 PMC4823177

[29] Wood R Bassett K Foerster V: PROS AND CONS OF 1.5 T MRI VERSUS 3.0 T MRI. Reference Source

[30] Coffey CE Lucke JF Saxton JA: Sex differences in brain aging. a quantitative magnetic resonance imaging study. *Arch Neurol.* 1998;55(2):169–179. 10.1001/archneur.55.2.169 9482358

[31] Grant MK Bobilev AM Pierce JE: Dataset 1 in: Structural brain abnormalities in 12 persons with aniridia. *F1000Research.* 2017 Data Source 10.12688/f1000research.11063.1 PMC5615777 29034075

