# Supplementary figures and images for: Structural brain abnormalities in 12 persons with aniridia
Source: F1000Res. 2017 Sep 1;6:255. Originally published 2017 Mar 13. [Version 2] doi: 10.12688/f1000research.11063.2 (PMC5615777; doi:10.12688/f1000research.11063.2)

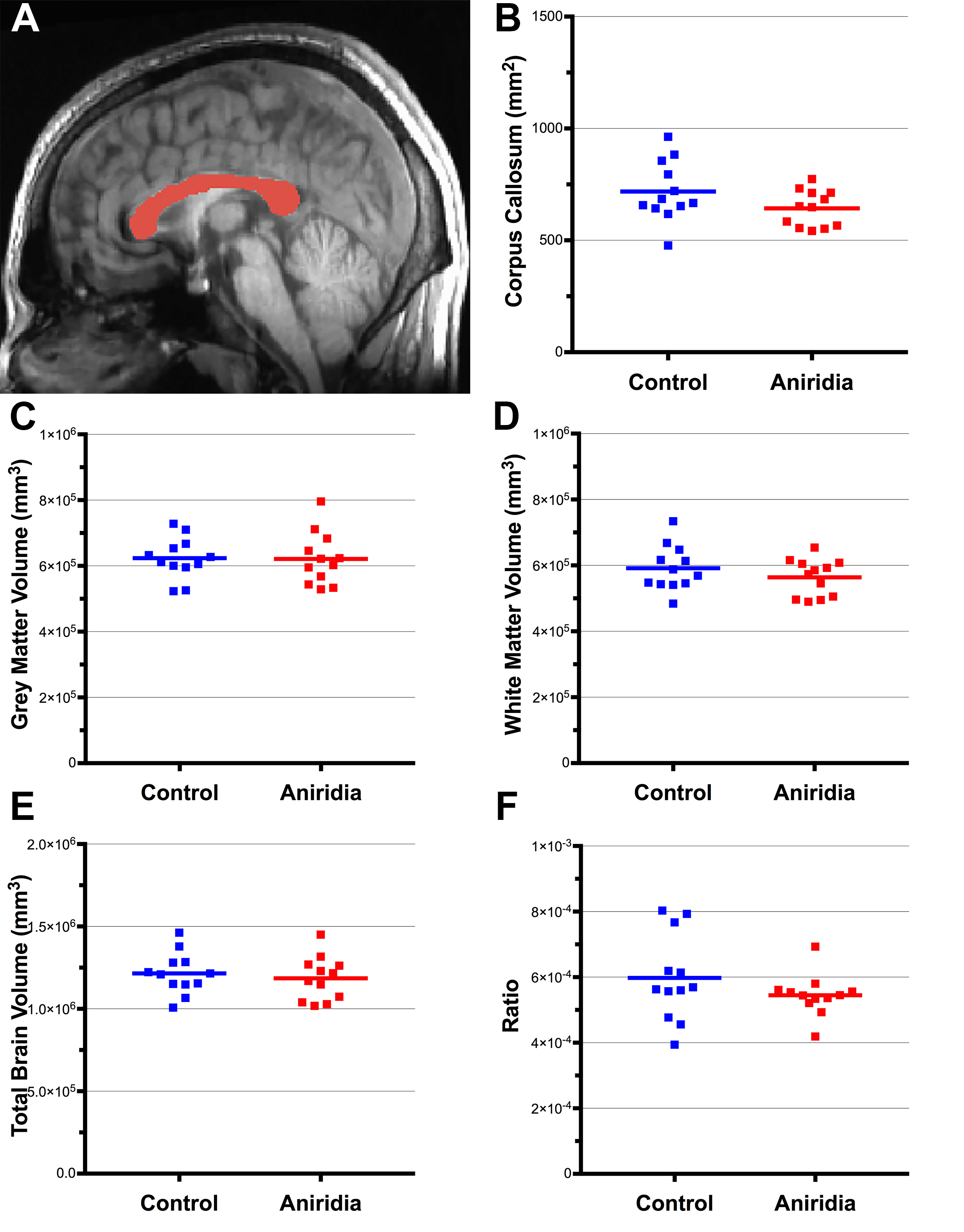

Supplement: Supplementary file 2 [file f1000research-6-13553-s0001.tgz › 89bc0485-f337-4530-8ea8-f963ee1aacef.tif]
