# Supplementary material for: Structural brain abnormalities in 12 persons with aniridia
Source: F1000Res. 2017 Sep 1;6:255. Originally published 2017 Mar 13. [Version 2] doi: 10.12688/f1000research.11063.2 (PMC5615777; doi:10.12688/f1000research.11063.2)
Supplement: Peer review history

# Peer review: Referee response for version 2

**DOI:** 10.5256/f1000research.13553.r25602

**Published:** 2017-09-26

**Authors:** Lutz Jäncke

**Affiliations:** 1 Department of Psychology (Neuropsychology) and INAPIC, University of Zurich, Zurich, Switzerland

The paper is now fine and the authors have nicely coped with the reviewer's suggestions and comments.

I have read this submission. I believe that I have an appropriate level of expertise to confirm that it is of an acceptable scientific standard.

# Peer review: Referee response for version 1

**DOI:** 10.5256/f1000research.11931.r21372

**Published:** 2017-04-18

**Authors:** Lutz Jäncke

**Affiliations:** 1 Department of Psychology (Neuropsychology) and INAPIC, University of Zurich, Zurich, Switzerland

This is an interesting and extremely important study reporting specific neuroanatomical features in patient group suffering from a rare disease affecting the visual system: aniridia. I have listed my comments and suggestions below.

Introduction

 The introduction is fine and discusses the current literature. However, I would suggest to improve a bit the study question. The study question as it is used in the current version is very broad and does not come to the point (e.g.,"The current study sought to investigate gross anatomical correlates of aniridia in a new population sample. Results from this study will serve as a comparison for previous studies, as well as contribute to what is known about the distribution of neuroanatomical phenotypes in the aniridia population as a whole."). I would suggest to include a few statements what this new study will add to the literature and what the "problems" of the older studies are? In addition, it could be helpful for the reader to learn the very new and maybe innovative aspects of this study.

Methods

 Subjects: Some additional information about the subjects could be helpful (cognitive ability, handedness, language lateralization). Some of these variables have been shown to be strongly related to the anatomical measures for which the authors are reporting the specific aniridia features.

 Statistics: It is not entirely clear how the authors did their between-group comparisons. Did they compare the patients only on a visual-descriptive basis or did they perform a statistical analysis (which I would suggest). When doing a statistical analysis, one could compare single subjects to the mean of the control group. Thus, it would be possible to test each patient against the mean of the healthy control group. Using this comparison it would also be possible to calculate effect size measures for each subject.

 By the way, did the use a priorly defined regions of interest?

Discussion

 The discussion is fine.

I have read this submission. I believe that I have an appropriate level of expertise to confirm that it is of an acceptable scientific standard, however I have significant reservations, as outlined above.

# Response

**Published:** 2017-08-23

**Authors:** Madison Grant

**Affiliations:** University of Georgia, USA

**Dr. Lutz Jäncke**, Department of Psychology (Neuropsychology) and INAPIC, University of Zurich, Zurich, Switzerland 

We thank Dr. Jäncke for the helpful comments and suggestions, which improved the manuscript. Our responses are listed below.

Introduction

- Improve study question
   
  - Introduction has been revised to improve, clarify, and expand the description of our study question.
- Suggest: to include a few statements what this new study will add to the literature and what the "problems" of the older studies are? In addition, it could be helpful for the reader to learn the very new and maybe innovative aspects of this study.
   
  - Introduction has been revised to include more information on new aspects for this study. In particular we have emphasized at the end of the “ *Introduction*” section that this study utilized a 3T magnet instead of a 1.5T, allowing for higher resolution when examining small structures such as the anterior and posterior commissure.  Additionally, we also clarified, at the end of the “ *Introduction*” section, that our study examined five structures shown to be abnormal in the aniridia population. Previous studies focused on only a subset of structures in each patient rather than all five.  By examining all five structures in a new population of aniridia patients, we have added to the collective knowledge on structural brain abnormalities present within the disorder.  

Methods

- Subjects: additional information: cognitive ability, handedness, language lateralization
   
  - We added handedness and predicted protein effect to Table 1. We do not have data on cognitive ability or language lateralization for our participants.
- Statistics: It is not entirely clear how the authors did their between-group comparisons. Did they compare the patients only on a visual-descriptive basis or did they perform a statistical analysis (which I would suggest). When doing a statistical analysis, one could compare single subjects to the mean of the control group. Thus, it would be possible to test each patient against the mean of the healthy control group. Using this comparison it would also be possible to calculate effect size measures for each subject.
   
  - For this study, the structural changes in Table 1 were concluded by two independent reviews of the MRI scans using a visual-descriptive basis.  The methods have been updated to clarify the visual-descriptive assessment. 
  - Volumetric analysis was conducted for the corpus callosum using Freesurfer software and results have been added to the manuscript (see supplementary Table 1 and supplementary Figure 1). Findings from this volumetric analysis confirmed our structural examination of the corpus callosum for all subjects.  We did not conduct quantitative volumetric analyses on the anterior and posterior commissures as these are relatively small structures and the plane of view was not consistent within all subjects. This is due to variation in slice location within each brain. Because of this we decided to rely on our visual-descriptive assessment of these two structures for our dataset. While not ideal, a visual-descriptive assessment of the anterior and posterior commissures is consistent with previous reports comparing these structures between PAX6 normal individuals and individuals with PAX6 mutations (e.g. Sisodiya et al. 2001; Abouzeid et al., 2009) and captures relative changes between these two groups.
- By the way, did the use a priorly defined regions of interest?
   
  - Yes, our Methods section (MRI data analysis) has been revised to state how we determined regions of interest.  We used literature review to define which structures would be examined, and had ourthe radiologist (JD) examined the MRI scans  for identification of additional regions of interest.

Discussion

The discussion is fine.

# Response

**Published:** 2017-09-17

**Authors:** Lutz Jäncke

**Affiliations:** Psychology Division Neuropsychology, University of Zurich, Switzerland

Thank you so much. Well done!

# Peer review: Referee response for version 1

**DOI:** 10.5256/f1000research.11931.r21598

**Published:** 2017-04-06

**Authors:** Veronica van Heyningen

**Affiliations:** 1 Institute of Ophthalmology, University College London, London, UK

This is an interesting report on structural brain changes in 12 aniridia patients examined by structural MRI studies, using 12 selected matching controls for comparison. The title and abstract convey details appropriately.

 I should say at the outset that I have no expertise in MRI methodology and cannot compare the techniques used here with those used elsewhere, including our own papers.

 It is clear, however, that MRI has advanced technically to a significant extent since the first set of papers was published in 2001-3. We need to know how directly comparable the results are. Certainly Sisodiya et al. in 2001 (ref 17) Mitchell et al. and 2003 (ref 18) used voxel-based morphometry and less powerful MRI measurements but showed similar findings with observed reduction or absence of the anterior commissure and olfactory bulb (ref 17) and of the pineal (ref 18). The current study does not mention study of the olfactory bulb. Interestingly olfactory function was also shown to be reduced and very occasionally absent in the first study (ref 17). Reduced interhemispheric transfer is thought to underlie the observed auditory processing problems reported in children with aniridia in ref 19. The current study reported in this paper does not undertake functional smell or auditory tests. Although what seems to be the same cohort, judging by the reported mutations, has been studied by functional fMRI by Pierce et al (ref 24). Surprisingly this overlapping author group (with the current paper) reports an apparently increased functional connectivity in the patients studied in the current paper. The earlier studies by other groups do not look at these functional connectivity changes. It is regrettable that the later studies, including Yogarajah et al (ref 23) did not follow this up.

 Otherwise the major findings are remarkably similar for this study and the previous studies to explore 12 or more cases. The controls in ref 17 and 18 are larger numbers (100) and not directly paired controls. The study in Ref 20 where the posterior commissure reduction was observed, only looked at the index case from three families with aniridia and PAX6 mutations. Reference 23 re-examines many of the same cases as Ref 17 and 18, but about a decade later and with more powerful MRI using surface-based morphometry. The reduced cortical measurements are more obvious in these older cases. The observed reduction of the optic chiasm reported here  in 7/12 cases is an interesting new finding, first suggested in the one of the three families studied by Abouzeid et al, ref 20.

 Minor comments

1. In the Introduction the PAX6 mutations are described as mostly “single nucleotide polymorphisms”. This is incorrect the changes are extremely rare variants. There are no polymorphisms of any note in the extremely highly conserved PAX6 gene.
2. It is a pity that the patient numbering is different in earlier fMRI work from this group (ref 24). Indeed it is not made clear that ref 24 studies the same cohort as observed in the current paper.
3. It would help to have the protein effects of the mutations in Table 1.
4. Can the olfactory bulb status be observed with the current cohort? It would be interesting to see that reported.

I have read this submission. I believe that I have an appropriate level of expertise to confirm that it is of an acceptable scientific standard.

# Response

**Published:** 2017-08-23

**Authors:** Madison Grant

**Affiliations:** University of Georgia, USA

**Reviewer: Veronica van Heyningen**, Institute of Ophthalmology, University College London, London, UK 

We thank Dr. van Heyningen for the helpful comments and suggestions, which improved the manuscript. Our responses are listed below.

Comments to the Author:

Minor comments

1. In the Introduction the PAX6 mutations are described as mostly “single nucleotide polymorphisms”. This is incorrect the changes are extremely rare variants. There are no polymorphisms of any note in the extremely highly conserved PAX6 gene.
  - The text was modified to read: “The majority of these variants are nonsense mutations, which lead to a premature termination codon, and are found across the *PAX6* locus.”
2. It is a pity that the patient numbering is different in earlier fMRI work from this group (ref 24). Indeed it is not made clear that ref 24 studies the same cohort as observed in the current paper.
  - Numbering of patients has been amended to reflect the same numbering used in the previous paper (ref 24).  We have additionally added a supplementary table (Supplementary Table 2) that matches DICOM file names to patient ID from this paper and previous papers using the same patient group.  Additionally, we have added a sentence in methods (Subjects) to state that this study and ref 24 used the same patients.
3. It would help to have the protein effects of the mutations in Table 1.
  - Protein effects have been added to Table 1.
4. Can the olfactory bulb status be observed with the current cohort? It would be interesting to see that reported.
  - While we agree that the olfactory bulb would be an interesting and important addition, we elected not to pursue this structure because constraints inherent to our data.  We have amended methods (MRI data analysis) to discuss explain why we decided not to investigate olfactory bulb in this patient sample.
